# Brain connectivity changes when comparing effects of subthalamic deep brain stimulation with levodopa treatment in Parkinson's disease

**DOI:** 10.1016/j.nicl.2018.05.006

**Published:** 2018-05-09

**Authors:** Karsten Mueller, Robert Jech, Filip Růžička, Štefan Holiga, Tommaso Ballarini, Ondrej Bezdicek, Harald E. Möller, Josef Vymazal, Evžen Růžička, Matthias L. Schroeter, Dušan Urgošík

**Affiliations:** a Max Planck Institute for Human Cognitive and Brain Sciences, Leipzig, Germany; b Department of Neurology and Center of Clinical Neuroscience, Charles University, First Faculty of Medicine and General University Hospital in Prague, Prague, Czech Republic; c Department of Stereotactic and Radiation Neurosurgery, Na Homolce Hospital, Prague, Czech Republic; d Clinic for Cognitive Neurology, University Hospital Leipzig, Germany

**Keywords:** Deep brain stimulation, Levodopa, Parkinson's disease, Resting state magnetic resonance imaging, Eigenvector centrality, Brain connectivity, Functional connectivity, Nexopathy, Subthalamic nucleus, STN

## Abstract

Levodopa and, later, deep brain stimulation (DBS) have become the mainstays of therapy for motor symptoms associated with Parkinson's disease (PD). Although these therapeutic options lead to similar clinical outcomes, the neural mechanisms underlying their efficacy are different. Therefore, investigating the differential effects of DBS and levodopa on functional brain architecture and associated motor improvement is of paramount interest. Namely, we expected changes in functional brain connectivity patterns when comparing levodopa treatment with DBS.

Clinical assessment and functional magnetic resonance imaging (fMRI) was performed before and after implanting electrodes for DBS in the subthalamic nucleus (STN) in 13 PD patients suffering from severe levodopa-induced motor fluctuations and peak-of-dose dyskinesia. All measurements were acquired in a within subject-design with and without levodopa treatment, and with and without DBS. Brain connectivity changes were computed using eigenvector centrality (EC) that offers a data-driven and parameter-free approach—similarly to Google's PageRank algorithm—revealing brain regions that have an increased connectivity to other regions that are highly connected, too. Both levodopa and DBS led to comparable improvement of motor symptoms as measured with the Unified Parkinson's Disease Rating Scale motor score (UPDRS-III). However, this similar therapeutic effect was underpinned by different connectivity modulations within the motor system. In particular, EC revealed a major increase of interconnectedness in the left and right motor cortex when comparing DBS to levodopa. This was accompanied by an increase of connectivity of these motor hubs with the thalamus and cerebellum.

We observed, for the first time, significant functional connectivity changes when comparing the effects of STN DBS and oral levodopa administration, revealing different treatment-specific mechanisms linked to clinical benefit in PD. Specifically, in contrast to levodopa treatment, STN DBS was associated with increased connectivity within the cortico-thalamo-cerebellar network. Moreover, given the favorable effects of STN DBS on motor complications, the changes in the patients' clinical profile might also contribute to connectivity changes associated with STN-DBS. Understanding the observed connectivity changes may be essential for enhancing the effectiveness of DBS treatment, and for better defining the pathophysiology of the disrupted motor network in PD.

## Highlights

• Functional MRI was done before and after implanting DBS electrodes in same patients.
• Impacts of DBS and levodopa administration on brain motor circuitry are different.
• Comparison between DBS and levodopa treatment shows a major connectivity increase.
• Treatment related connectivity changes can be disentangled from electrode implantation.

## 1 Introduction

Levodopa has been the mainstay of symptomatic therapy for Parkinson's disease (PD) for the last five decades (Birkmayer and Hornykiewicz, 1961). However, it is associated with the development of motor fluctuations and dyskinesias, in particular after several years of treatment. To counteract these debilitating late complications, deep brain stimulation (DBS) has been introduced as an alternative therapy (Benabid et al., 1991; Odekerken et al., 2013; Smith et al., 2012). While processes needed for levodopa to exert its antiparkinsonian effect are understood relatively well (Poewe et al., 2010), the physiological mechanisms leading to effectiveness of DBS still remain to be clarified (Chiken and Nambu, 2016). Investigating differential mechanisms of these treatment options is therefore of great interest for deepening the understanding of PD pathophysiology and laying the groundwork for further advances in development of PD therapies. Here, for the first time, we addressed the comparison between the effect of levodopa medication and DBS of the subthalamic nucleus (STN) on functional brain connectivity in a group of 13 PD patients examined before and after electrode implantation. We focused our analyses on changes in brain connectivity, in accordance with recent frameworks conceptualizing neurodegenerative diseases as nexopathies, where pathology and treatment are characterized by specific changes in brain connectivity (Warren et al., 2012).

To identify brain connectivity changes, we used resting-state functional magnetic resonance imaging (fMRI) combined with graph-theory approaches (Bullmore and Sporns, 2009, Bullmore and Sporns, 2012), namely a data-driven and parameter-free analysis technique called Eigenvector Centrality (EC) mapping (Lohmann et al., 2010). This method detects central hubs within a brain network using an algorithm similar to Google's PageRank algorithm. In the same way the PageRank algorithm highlights websites that are most often linked with other highly connected sites (Brin and Page, 1998), we were interested in brain regions that are strongly connected to other highly connected areas. Using EC mapping, we investigated how switching the treatment type from dopaminergic medication to DBS in the STN modulated central hubs of brain connectivity in PD. Here, maps of EC differences show changes of the degree of regional brain interconnectedness between both treatment states. Thus, an increased EC of a region reflects an increased connectivity to other highly connected regions in the brain. However, while EC indicates connectedness, it does not yield information on the connections themselves, that is, it does not show which regions are connected to the reference region. Therefore, seed-based correlation analyses were performed in addition to the EC analysis using regions with maximum EC differences as reference regions (Taubert et al., 2011).

By identifying brain connectivity differences between the two best-established and clinically most-successful treatment approaches in PD, levodopa and DBS, our study aims at characterizing treatment-specific brain mechanisms in PD leading to an improved clinical outcome. Previous studies have investigated the effects of either levodopa or STN DBS on brain connectivity, but a direct comparison of the two approaches is still missing. A recent review summarized the effects of dopaminergic medication on functional brain connectivity comparing PD patients under both medicated and unmedicated states (Tahmasian et al., 2015). Despite the wide methodological and clinical heterogeneity, dopaminergic therapy was generally able to turn off the aberrant brain connectivity observed in the unmedicated state, thus having a normalizing effect (Tahmasian et al., 2015). Concerning STN DBS, fewer studies have investigated its effect on brain functional connectivity. Remarkably, Kahan et al. (2014) showed that STN DBS modulates the effective connectivity within the entire cortico-striato-thalamo-cortical loop. This is in line with the view of DBS as a modulator of distributed brain networks, rather than isolated brain regions (Fox et al., 2014; Lozano and Lipsman, 2013). Additionally, Horn et al. (2017) combined data from PD patients implanted with STN DBS with human connectome data from healthy subjects to investigate which functional and structural connectivity patterns are predictive of clinically effective STN DBS. They reported that a negative functional correlation between the STN and primary motor cortex was predictive for a better treatment response (Horn et al., 2017). As we showed previously, levodopa increased connectivity in the cerebellum and brainstem (Jech et al., 2013), whereas DBS in the STN enhanced connectivity in the premotor cortex in PD as measured with EC mapping (Holiga et al., 2015; Mueller et al., 2013). Remarkably, the degree of connectedness in this region correlated negatively with clinical symptoms as measured with the Unified Parkinson's Disease Rating Scale (UPDRS) motor score.

In the current work, we directly compare the effects of DBS in the STN and of levodopa on brain connectivity in PD in a within-subject study design. Remarkably, the underlying therapeutic mechanisms of levodopa and DBS are fundamentally different. The former is a dopamine precursor, whose therapeutic effect is mainly mediated by the increase of striatal dopamine availability (LeWitt, 2015). The latter, instead, acts through a direct high-frequency electrical stimulation of the STN, inducing a complex modulatory effect further distributed to other structures which the STN is connect to (Jech et al., 2001; McIntyre et al., 2004) with an endogenous increase of dopamine release (Strafella et al., 2003). The main aim of this study was, thus, to compare the STN DBS and levodopa treatments for PD to gain a better understanding of their functioning at the brain level. Specifically, we focused on the study of the brain functional architecture that has been proved to be a candidate in vivo marker for the study of neurodegenerative diseases (Warren et al., 2012) and for monitoring treatment-related changes in PD (Fox et al., 2014; Tahmasian et al., 2015). Based on previous studies and on the knowledge concerning different underlying mechanisms (Holiga et al., 2015; Horn et al., 2017; Jech et al., 2013; Kahan et al., 2014; Mueller et al., 2013; Tahmasian et al., 2015), we hypothesize that levodopa and STN DBS would lead to different connectivity reorganization within the motor network. Specifically, we expect that the STN DBS will affect the functional connectivity of brain structures that are connected in the hyperdirect and indirect pathways in the motor network (Brunenberg et al., 2012; Jahanshahi et al., 2015; McIntyre and Hahn, 2010). Purposely, we studied unilateral DBS to investigate if it is associated with a lateralized or bilateral effect on brain connectivity (Arai et al., 2008; Kumar et al., 1999), in contrast with levodopa for which we expected a systemic effect. As a disposition of PD patients to react on levodopa with dyskinesias may specifically affect the functional connectivity (Herz et al., 2016), only patients expressing peak of dose chorea were included in the study.

## 2 Methods

### 2.1 Patient cohort

Resting-state fMRI was performed in a set of 13 PD patients in advanced stage (equivalent akinetic/rigid type, Hoehn-Yahr stages II-III, 11 males, age 52.8 ± 6.9 years, disease duration 12.6 ± 2.7 years, levodopa treatment duration 9.5 ± 3.1 years) in a within-subject study design. All patients suffered from unbearable motor complications (wearing off, motor fluctuations and peak of dose dyskinesias) considered as inclusion criteria for the STN DBS treatment. A detailed description of the patient cohort is shown in Table 1. All procedures conformed to the Declaration of Helsinki. The study protocol was approved by the Ethics Committee of the General University Hospital in Prague, Czech Republic. All patients gave informed written consent.

**Table 1:** List of patients and demographical details.

ID	Sex	Age	PD dur	PD treat	U *PRE*_*OFF*_	U *PRE*_*ON*_	U *POST*_*OFF*_	U *POST*_*ON*_
1	M	63	15	13	21	5	24	9
2	M	53	11	7	44	9	32	13
3	M	53	12	10	36	10	28	12
4	M	46	15	7	47	20	28	8
5	M	64	13	8	31	10	21	12
6	M	53	12	9	43	9	24	6
7	M	49	13	12	64	18	39	21
8	M	55	12	9	46	21	24	8
9	M	60	14	14	18	8	19	13
10	F	42	9	6	33	7	27	10
11	M	55	19	15	35	4	14	3
12	M	43	9	7	34	15	33	15
13	F	50	10	6	19	6	17	7

*U = Unified Parkinson's Disease Rating Scale (UPDRS)-III score; *PRE* refers to clinical assessment prior to the implantation of the DBS electrodes, and *POST* refers to the examinations after the DBS surgery. The subscript notation *ON* and *OFF* denotes the state of medication. For the *PRE* sessions, *ON* and *OFF* refers to the levodopa treatment, and for the *POST* sessions, *ON* and *OFF* refers to DBS treatment; age, PD disease duration (PD dur), and PD treatment duration (PD treat) is shown in years.

### 2.2 Design of the study

For each patient, clinical assessment and MRI was performed in four different sessions: *PRE*_*OFF*_, *PRE*_*ON*_, and *POST*_*OFF*_, *POST*_*ON*_. In our notation, *PRE* will always refer to clinical assessment and imaging sessions *prior* to the implantation of the DBS electrodes, and *POST* will always refer to the examinations *after* the DBS surgery. In short, *PRE* and *POST* stands for the *treatment method*. The subscript notation *ON* and *OFF* denotes the *treatment state*. For the *PRE* sessions, *ON* and *OFF* refer to the levodopa treatment, i.e. *PRE*_*OFF*_ stands for the session including clinical assessment and imaging without levodopa medication. For the *POST* sessions, *ON* and *OFF* refer to DBS treatment, i.e. *POST*_*OFF*_ is written for investigations and imaging after DBS surgery but without using the DBS electrodes.

The first two levodopa-related examinations *PRE*_*OFF*_ and *PRE*_*ON*_ were performed 18.2 ± 18.1 days prior to implantation of the DBS electrodes. Four days before all measurements, dopamine agonists were substituted by equivalent doses of levodopa in each patient (Tomlinson et al., 2010). Other anti-PD medication (selegiline, amantadine, anticholinergics) was suspended. After an overnight withdrawal of levodopa (at least 12 h), clinical and MRI data were obtained with the *PRE*_*OFF*_ session. Clinical and imaging assessment with medication was performed in the *PRE*_*ON*_ session after administration of 250/50 mg of levodopa/carbidopa. These patients were scanned soon after beginning of their *ON* state in the period before appearance of dyskinesias. This has been documented by a clinical observation performed during each MRI session.

Implantation of the DBS system was performed separately in two surgeries according previously described procedures (Jech et al., 2001). The first surgery was carried out in awake state, during which the patient with attached Leksell stereotactic frame and motor microdriver underwent electrophysiology mapping of the subthalamic area with five parallel microelectrodes. Then, the intraoperative stimulation by macroelectrode was performed in a region with a neuronal signal typical for STN to confirm clinical benefit and to monitor potential adverse effects of DBS. The macroelectrode was eventually replaced by the permanent electrode (type 3389, Medtronic, MN) connected to external leads.

Both DBS sessions *POST*_*OFF*_ and *POST*_*ON*_ were scheduled within 1–3 days after the first surgery when the electrodes were externalized and connected to an external stimulator working in bipolar mode (Dual Screen 3628, Medtronic, Minneapolis, MN). Clinical assessment and MRI was performed in both post-surgery sessions *POST*_*OFF*_ and *POST*_*ON*_, i.e. without and with STN DBS. Note that clinical assessment was achieved in the *POST*_*ON*_ session using bilateral STN DBS while functional imaging was performed in two conditions, *POST*_*ON_left*_ and *POST*_*ON_right*_, using unilateral left STN DBS and unilateral right STN DBS with bipolar mode of stimulation. The DBS parameters were kept below threshold for dyskinesias and above the threshold for rigidity/akinesa. This was achieved in all included patients as dyskinesias were never observed with the STN DBS during the MRI acquisition. To show potential variability of the clinical benefit, correlation analysis was performed between the stimulation amplitude and the intensity estimated by a previously published formula (Jech et al., 2006). Individual stimulation parameters are shown in Supplementary Table S3. Moreover, since the therapeutic effect of STN DBS might last even after switching off the neurostimulator, the *POST*_*OFF*_ and *POST*_*ON*_ conditions were randomized across the group to avoid order effects. Similarly, the order of *POST*_*ON_left*_ and *POST*_*OFF_right*_ was alternated. Implantation of the internal pulse generator (Kinetra or PC) in the subclavial region was done under general anesthesia one day after the MRI acquisition.

### 2.3 Clinical data analysis

PD symptoms were assessed with the UPDRS motor score (part III) in all four sessions *PRE*_*OFF*_, *PRE*_*ON*_, and *POST*_*OFF*_, *POST*_*ON*_. Clinical data were analyzed with a repeated measure ANOVA procedure including the factors *treatment method* (*PRE* vs. *POST*) and *treatment state* (*OFF* vs. *ON*). Post hoc analyses were conducted by calculating five two-tailed paired Student's *t*-tests including Bonferroni correction for multiple testing using a final significance level of α = 0.0083. Subsequently, data are generally reported as mean ± standard deviation. Moreover, the so-called microlesion effect (Holiga et al., 2015; Jech et al., 2012) following implantation of DBS electrodes might cause a transient improvement in the post-surgery *POST*_*OFF*_ condition compared with the pre-surgery *PRE*_*OFF*_ condition. This creates an imbalance when comparing the main effects of both treatments separately. Thus, we also used *PRE*_*OFF*_ as a common baseline for the no treatment (*OFF*) condition, and compared the effects ‘*PRE*_*OFF*_ vs. *PRE*_*ON*_’ and ‘*PRE*_*OFF*_ vs. *POST*_*ON*_’.

### 2.4 Image acquisition

Functional MRI data were obtained using a 1.5-T MAGNETOM Symphony scanner (Siemens Healthcare, Erlangen, Germany) and *T*_2_*-weighted gradient-echo echo-planar imaging (EPI) (repetition time, *TR* = 3 s; echo time, *TE* = 51 ms). For every patient, functional data were obtained in all examination sessions. Two fMRI scans were obtained without and with antiparkinsonian medication before DBS surgery (*PRE*_*OFF*_, *PRE*_*ON*_), and three fMRI scans were measured after implantation of the DBS electrodes without simulation, with unilateral left DBS, and with unilateral right DBS (*POST*_*OFF*_, *POST*_*ON_left*_, *POST*_*ON_right*_). Each data set was acquired with 200 functional volumes and 31 axial slices (thickness = 3 mm, gap = 1 mm) with a nominal in-plane resolution of 3 × 3 mm^2^ covering the whole brain. Patients were asked to keep still, awake, and look at a fixation cross on a projection screen.

For registration purposes, *T*_*1*_-weighted images were obtained using a magnetization-prepared rapid gradient echo (MP-RAGE) sequence (*TR* = 2140 ms; inversion time, *TI* = 1100 ms, *TE* = 3.93 ms, *flip angle* = 15°) before and after DBS surgery. The MRI scans were performed according to previously defined technical precautions considering the potential hazard in patients with intracerebral electrodes (Jech et al., 2001). The main concern performing MRI studies with DBS electrodes in place (especially with impulse generator turned on) is related to potential overheating induced by the scanner radio frequency (Rezai et al., 2004). However, it has been demonstrated that both structural and functional MRI assessment, especially at 1.5 T, are safe, even with active DBS system (Bronstein et al., 2011; Carmichael et al., 2007). To minimize any potential danger, the *POST*_*ON*_ scans were split into two different sessions (*POST*_*ON_left*_ and *POST*_*ON_right*_).

Position of each electrode contact in the STN was identified on T1-weighted images in native space of each patient according to previously described procedure (Ruzicka et al., 2012). Briefly, the x-coordinate of each contact was measured manually from the wall of the third ventricle, whereas the y- and z-coordinates were measured from the mid-commissural point. Correlation analysis was performed between clinical improvement assessed by UPDRS-III and the x,y,z-coordinates of the distal (−) and proximal (+) active contact in both hemispheres. Individual contact positions are reported in Supplementary Table S3.

### 2.5 Image pre-processing

All resting-state fMRI data sets were processed using SPM8 (Wellcome Trust Centre for Neuroimaging, UCL, London, UK) and Matlab® (The MathWorks Inc., Natick, MA). Standard pre-processing included realignment, slice-time correction, normalization to the Montreal Neurological Institute (MNI) space based on the unified segmentation approach (Ashburner and Friston, 2005), and spatial filtering using a Gaussian kernel with 8-mm full width at half maximum.

### 2.6 Eigenvector centrality analysis

Eigenvector centrality (EC) was computed using the Lipsia software package (Lohmann et al., 2001). For obtaining the EC, a similarity matrix was computed including Pearson's correlation coefficient between all resting-state fMRI time courses. In order to use a similarity matrix with only positive numbers, we added the number one to all correlations (the EC ‘add’ approach, also implemented in (Wink et al., 2012). To further assess the effect of negative correlations, we also used two other approaches using the absolute values of the correlation values (EC ‘abs’), and setting negative correlation values to zero (EC ‘pos’) before computing the EC (Lohmann et al., 2010). According to the theorem of Peron and Frobenius, a similarity matrix with positive entries has a unique real largest eigenvalue, and the corresponding eigenvector has strictly positive components (Frobenius, 1912; Perron, 1907). Finally, the EC map was generated using the *i-*th component of this eigenvector to obtain the EC value for voxel *i*.

After computing EC maps for all patients and all experimental conditions (*PRE*_*OFF*_, *PRE*_*ON*_, and *POST*_*OFF*_, *POST*_*ON_left*_, *POST*_*ON_right*_), a group analysis was performed using the general linear model with a flexible factorial design including all 13⋅5 = 65 EC maps (5 columns defining all experimental conditions and 13 columns defining the subject factor). Various tests were performed in order to check for EC differences using contrast vectors. Here we tested for EC differences between DBS and levodopa therapy with a *PRE-POST*-comparison between both *ON*-states *POST*_*ON_left*_ and *PRE*_*ON*_. In particular, we looked at the difference *POST*_*ON_left*_*−PRE*_*ON*_. To investigate whether this EC difference is induced by general *PRE-POST*-effects as microlesion due to the surgery procedure (Jech et al., 2012), we also computed the *PRE-POST*-contrast for both *OFF*-conditions *POST*_*OFF*_*−PRE*_*OFF*_. Finally, to show that our treatment-related EC differences are really induced by the different treatment types, and not just an effect of microlesion and/or other *PRE-POST*-effects, we computed the interaction of both factors *treatment method* (*PRE/POST*) and *treatment state* (*ON/OFF*) using the difference of difference (*POST*_*ON_left*_*−PRE*_*ON*_)−(*POST*_*OFF*_*−PRE*_*OFF*_). To investigate both left and right unilateral DBS, we repeated all analysis with *POST*_*ON_right*_ instead of *POST*_*ON_left*_. Finally, we also used a weighted contrast to compare the sum of EC of both *ON*-conditions *POST*_*ON_right*_ + *POST*_*ON_left*_ with *PRE*_*ON,*_ and we will shortly write *POST*_*ON*_*−PRE*_*ON*_ to denote the EC difference. Here, we also computed the interaction contrast (*POST*_*ON*_*−PRE*_*ON*_)−(*POST*_*OFF*_−*PRE*_*OFF*_). Resulting statistical parametric maps were corrected for multiple comparisons using the false discovery rate (FDR) approach with *p* < 0.05. Note that all computations were performed with all three approaches of dealing with negative correlations when computing the EC (see ‘add’, ‘abs’, and ‘pos’ described in the previous paragraph).

### 2.7 Seed-based correlations

In order to detect brain regions which contribute to EC differences investigated above, seed-based correlation analyses were performed in addition to EC mapping. Seed regions were defined by clusters obtained with significant EC differences between the *PRE* and the *POST* session using the contrast *POST*_*ON*_*−PRE*_*ON*_ described in the previous section. Using this contrast, two seed regions were defined in the left and right primary motor cortex. Using the average BOLD signal in both seed regions, correlations maps were generated for all five fMRI data sets collected for the five experimental conditions for each subject. These 65 (=5 × 13) correlation maps were fed into a general linear model using the same flexible factorial design used to detect EC differences as described above. We also computed the same contrasts *POST*_*ON_left*_*−PRE*_*ON*_ and *POST*_*OFF*_*−PRE*_*OFF*_, and the difference of difference (*POST*_*ON_left*_*−PRE*_*ON*_)−(*POST*_*OFF*_*−PRE*_*OFF*_). Then, the same analysis was repeated with *POST*_*ON_right*_ instead of *POST*_*ON_left*_. Significant clusters were detected using *p* < 0.05 (FDR-corrected). All contrasts were computed for both seed regions.

### 2.8 Geometric distortions

A major issue in fMRI with DBS is the occurrence of geometric distortions and drop-out of the EPI signal in the vicinity of the electrodes, particularly near the skull where electrodes are connected to the extension leads. Therefore, to prevent potential false-positive results, voxels exhibiting severe magnetic susceptibility artifacts caused by the presence of the DBS apparatus in the static magnetic field were excluded from the search space. In particular, a voxel-wise intensity threshold of 40% was employed to obtain an individual post-surgery mask. Individual masks were combined across patients with the logical “and” operation. In addition, EC calculations were restricted to regions masked in a search space comprising the motor system specifically (premotor, motor and sensory cortex, basal ganglia, brainstem, and cerebellum) based on the WFUPickAtlas. Here, we used exactly the same mask as used in preceding articles (Holiga et al., 2015; Jech et al., 2013). The final mask was then formed as a conjunction between the anatomical search space and the average intensity mask (i.e. only the voxels present in both images were used for further analyses).

### 2.9 Motion effects

Generally, head motion might bias the connectivity analysis and, finally, the EC values due to motion-induced signal fluctuations. This could be a particular problem if the degree of motion-related artifacts would vary between the individual scanning sessions, for example, as a consequence of treatment. Therefore, we checked for differences in head motion between all scanning sessions by computing the framewise displacement (FD) as introduced in (Power et al., 2012). As an input, we used the translational and rotational motion parameters obtained by SPM's motion correction. For the whole series of 200 functional images, motion between volumes was characterized using 199 FD values for each session and subject. Finally, for each session and subject, all FD time courses were characterized by the mean FD, the maximum FD, the maximum FD after eliminating the largest 5% of the FD values, and the number of FD values exceeding 2 mm.

## 3 Results

### 3.1 Clinical data

**Fig. 1:**
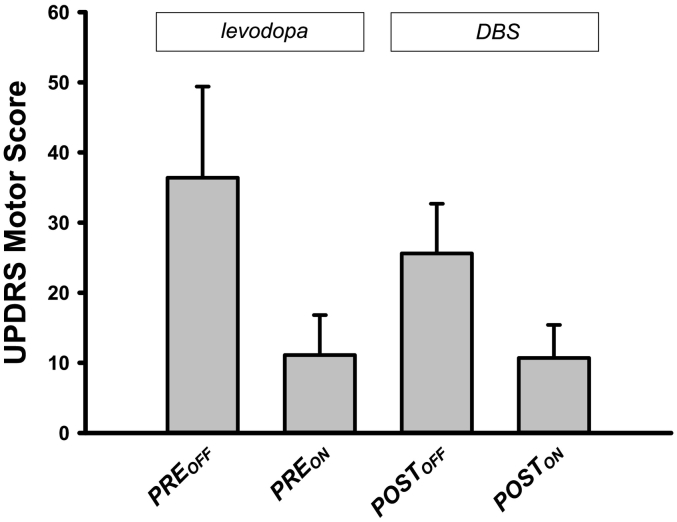
Motor symptoms of Parkinson's disease before (*PRE*) and after (*POST*) implantation of deep brain stimulation (DBS) electrodes as measured with the Unified Parkinson's Disease Rating Scale (UPDRS) motor score (part III). The subscript notation *ON* and *OFF* denotes the state of medication. For the *PRE* sessions, *ON* and *OFF* refer to the levodopa treatment, and for the *POST* sessions, *ON* and *OFF* refer to DBS treatment. The bars indicate mean values (across patients) ± standard deviation.

Fig. 1 illustrates clinical PD symptoms before (*PRE*) and after (*POST*) DBS implantation as measured with the UPDRS-III (motor) score. Scores are illustrated with or without respective treatment, levodopa or DBS. The motor symptoms were strongest in the levodopa off condition before DBS implantation. After DBS implantation without stimulation (*POST*_*OFF*_) clinical symptoms were slightly reduced. Bilateral STN DBS stimulation (*POST*_*ON*_) reduced motor symptoms in a similar manner as levodopa before surgery.

Effects of both factors *treatment method* (*PRE* vs. *POST* electrode implantation) and *treatment state* (*OFF* vs. *ON* for levodopa and DBS treatment) were statistically investigated with a repeated measure ANOVA procedure. The ANOVA demonstrated a significant main effect for the factor *treatment method* comparing all scores before and after the implantation of the DBS electrodes (*PRE* vs. *POST*) (*F* = 8.7, *df* = 1, *p* = 0.012), and for the factor *treatment state* (*ON* vs. *OFF*) comparing the scores with and without levodopa and DBS treatment (*F* = 123.4, *df* = 1, *p* < 0.001). We also obtained a significant interaction between both factors (*F* = 22.6, *df* = 1, *p* < 0.001). Post-hoc analyses with two-tailed paired Student's *t*-tests revealed treatment effects for levodopa treatment (*PRE*_*OFF*_ vs. *PRE*_*ON*_) (*T* = 9.3, *df* = 12, *p* < 0.001), and for DBS (*POST*_*OFF*_ vs. *POST*_*ON*_) (*T* = 12.2, *df* = 12, *p* < 0.001), with higher effects for levodopa (25.3 ± 9.9) than DBS (14.8 ± 4.4; *T* = 4.7, *df* = 12, *p* < 0.001). Note that the significant interaction effect between both factors *treatment method* (*PRE* vs. *POST*) and *treatment state* (*OFF* vs. *ON*) was caused by differences in the *OFF* condition, with reduced clinical symptoms in *POST*_*OFF*_ than *PRE*_*OFF*_ (*T* = 4.1, *df* = 12, *p* < 0.002), whereas there were no significant differences between clinical symptoms after both treatment approaches in the *ON* condition (*T* = 0.2, *df* = 12, *p* > 0.8).

When using *PRE*_*OFF*_ as a baseline for the no treatment (*OFF*) condition, a comparison between the effect ‘*PRE*_*OFF*_ vs. *PRE*_*ON*_’ (25.3 ± 9.9) and the effect of ‘*PRE*_*OFF*_ vs. *POST*_*ON*_’ (25.7 ± 12.0) did not yield a significant difference (*T* = −0.2, *df* = 12, *p* > 0.8). As revealed by subsequent analyses, there was no correlation of clinical improvement with the position of the active contact of the implanted electrode or with the stimulation parameters used in the STN DBS.

### 3.2 Eigenvector Centrality analysis

**Fig. 2:**
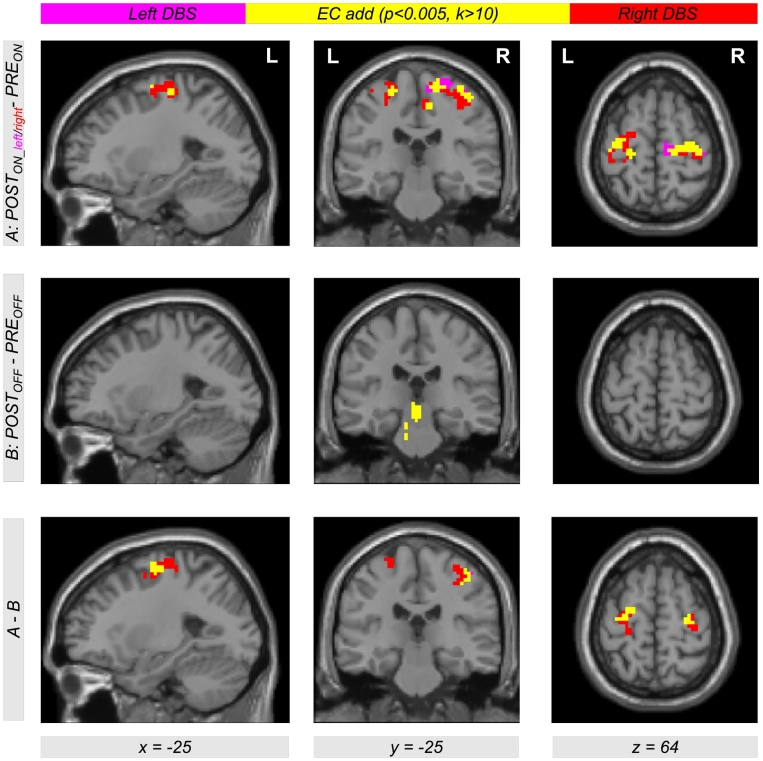
Differential effects of levodopa medication and deep brain stimulation (DBS) in the subthalamic nucleus (STN) on functional connectivity in motor networks affected by Parkinson's disease. The upper row shows Eigenvector Centrality (EC) differences between both treatment states when comparing levodopa medication (*PRE*_*ON*_) with DBS of the left and right STN (*POST*_*ON_left*_ and *POST*_*ON_right*_). Using the contrasts *POST*_*ON_left*_*−PRE*_*ON*_ and *POST*_*ON_right*_*−PRE*_*ON*_ (color-coded in magenta and red, respectively; overlap in yellow), we found increased EC with both left and right unilateral DBS compared with levodopa medication (see Table 2 for coordinates, cluster sizes, and statistical values). The second row shows the result for the same analysis comparing both *OFF*-conditions using the contrast *POST*_*OFF*_*−PRE*_*OFF*_. The bottom row shows the interaction between both factors *PRE/POST* and *OFF/ON* using the contrast including the difference of difference (*POST*_*ON_left*_*−PRE*_*ON*_)−(*POST*_*OFF*_*−PRE*_*OFF*_) and (*POST*_*ON_right*_*−PRE*_*ON*_)−(*POST*_*OFF*_*−PRE*_*OFF*_) (color-coded in magenta and red, respectively; overlap in yellow). The figure shows EC values with the ‘add’ approach (for the other approaches ‘pos’ and ‘abs’, see Figs. S1 and S2 in the Supplementary material).

**Fig. 3:**
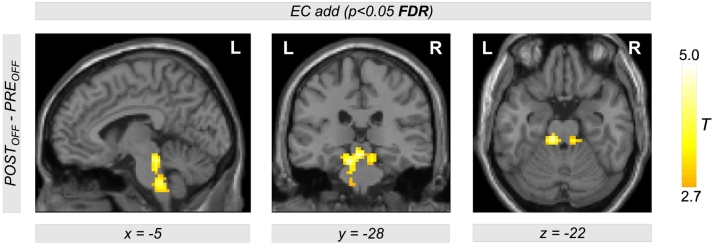
Eigenvector Centrality (EC) increase in the brain stem when comparing both *OFF*-states before and after DBS surgery. The contrast *POST*_*OFF*_−*PRE*_*OFF*_ is describing the microlesion effect (see Fig. 3c in (Holiga et al., 2015)).

**Fig. S1:**
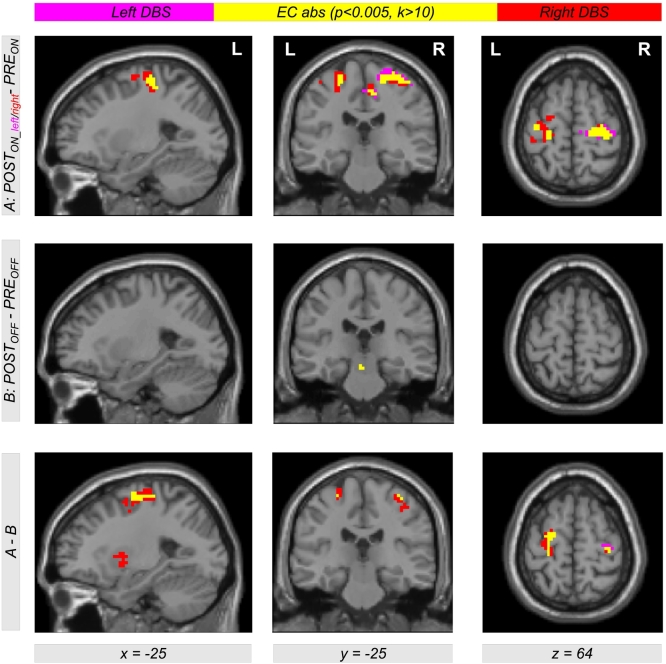
Differential effects of levodopa medication and deep brain stimulation (DBS) in the subthalamic nucleus (STN) on functional connectivity in motor networks affected by Parkinson's disease. The figure has the same content as Fig. 2 using the ‘abs’ approach instead of the ‘add’ approach for computing the EC.

Comparing EC during levodopa treatment to EC during unilateral left and right DBS using the contrasts *POST*_*ON_left*_−*PRE*_*ON*_ and *POST*_*ON_right*_−*PRE*_*ON*_, clusters of significant EC increase for both left and right DBS were obtained in the left and right motor cortex. Table 2 provides a list of coordinates, cluster sizes, *T*- and *FDR*-corrected *p*-values. The upper row of Fig. 2 shows the contrasts *POST*_*ON_left*_−*PRE*_*ON*_ (color-coded in magenta) and *POST*_*ON_right*_−*PRE*_*ON*_ (color-coded in red). Interestingly, we obtained a large overlap (color-coded in yellow) with both contrasts *POST*_*ON_left*_−*PRE*_*ON*_ and *POST*_*ON_right*_−*PRE*_*ON*_. Thus, comparing left and right STN DBS, we obtained EC differences in same brain regions in the motor cortex within both hemispheres when comparing both *ON*-states of unilateral DBS and levodopa treatment. The middle row of Fig. 2 shows EC differences between both *OFF*-states using the contrast *POST*_*OFF*_−*PRE*_*OFF*_ (microlesion effect). Here, we did not find an EC increase in the motor cortex but in the brain stem (Fig. 3). When testing the interaction effect between treatment method and state, i.e. (*POST*_*ON_left*_−*PRE*_*ON*_)−(*POST*_*OFF*_−*PRE*_*OFF*_) and (*POST*_*ON_right*_−*PRE*_*ON*_)−(*POST*_*OFF*_−*PRE*_*OFF*_), we obtained significant EC differences in the motor cortex with both left and right unilateral STN DBS (Fig. 2, bottom row, color-coded in magenta and red, respectively) compared with levodopa treatment. Note that all results shown in Fig. 2 were obtained computing the EC using the ‘add’ approach. However, we obtained same results using the ‘pos’ and the ‘abs’ approach as shown in Fig. S1, Fig. S2 in the supplement.

**Fig. 4:**
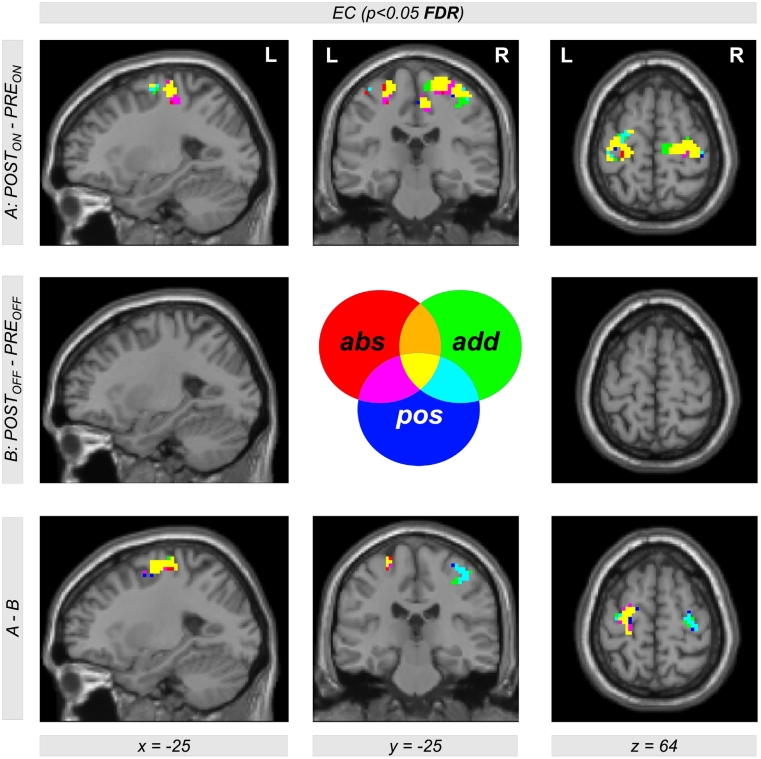
Eigenvector Centrality (EC) differences between both treatment states when comparing levodopa medication (*PRE*_*ON*_) with both left and right STN DBS using a weighted contrast (*POST*_*ON*_ = *POST*_*ON_right*_ + *POST*_*ON_left*_). The top row shows a color-coded map of EC differences with the contrast *POST*_*ON*_−*PRE*_*ON*_ using all three approaches of EC computation (‘abs’, ‘add’ and ‘pos’). Interestingly, there is a large overlap of EC computed with all three approaches (color-coded in yellow) indicating that EC differences are dominated by positive correlations between BOLD time courses. The middle row shows EC differences in the *OFF*-conditions using the contrast *POST*_*OFF*_*−PRE*_*OFF*_ (see also middle row of Fig. 2). The bottom row shows the interaction between both factors *PRE/POST* and *OFF/ON* using the interaction contrast including the difference of difference (*POST*_*ON*_*−PRE*_*ON*_)−(*POST*_*OFF*_*−PRE*_*OFF*_). Significant clusters were obtained with *p* < 0.05 using correction of multiple comparisons with the false discovery rate (FDR) approach (see Table 3 including coordinates, cluster sizes, and statistical values for the ‘add’ approach).

Due to the similar effect of using the left and the right unilateral STN DBS onto EC differences between DBS and levodopa treatment, we also used the contrast (*POST*_*ON_right*_ + *POST*_*ON_left*_)−*PRE*_*ON*_ (also written as *POST*_*ON*_−*PRE*_*ON*_) within our flexible factorial model. Using this contrast, we found significant EC differences in the left and right motor cortex with all three approaches of EC computation (‘ads’, ‘add’, and ‘pos’, Fig. 4). Thus, STN DBS, as compared to levodopa medication, increases the correlation between BOLD time courses of the motor cortex and other parts of the brain. Interestingly, this result is also present when computing the interaction between both factors *treatment method* (*PRE/POST*) and *treatment state* (*ON/OFF*) using the difference of difference (*POST*_*ON*_*−PRE*_*ON*_)−(*POST*_*OFF*_−*PRE*_*OFF*_) (Fig. 4, bottom row). This shows that our finding is really related to the different treatment approaches and not just induced by microlesion and/or by other *PRE-POST*-effects. All results were significant with *p* < 0.05 correcting for multiple comparisons using the FDR approach (Table 3 lists coordinates, cluster sizes, *T*- and *p*-values for the analysis computing EC using the ‘add’ approach).

**Table 2:** List of clusters showing an increased EC in the left and the right motor cortex with the ‘add’ approach comparing unilateral left and right DBS with levodopa medication using the contrasts *POST*_*ON_left*_*−PRE*_*ON*_ and *POST*_*ON_right*_*−PRE*_*ON*_⁎

*POST*_*ON_left*_―*PRE*_*ON*_							*POST*_*ON_right*_―*PRE*_*ON*_						
*x*	*y*	*z*	*T*	*Z*	*k*	*p*_*FDR-corr*_	*x*	*y*	*z*	*T*	*Z*	*k*	*p*_*FDR-corr*_
**30**	**-16**	**67**	**4.63**	**4.19**	**173**	**0.002**	**30**	**-19**	**64**	**4.70**	**4.24**	**183**	**0.001**
9	-28	49	4.40	4.01			15	-22	67	3.92	3.63		
24	-28	73	4.34	3.97			39	-25	61	3.74	3.49		
-36	-16	64	4.43	4.04	46	0.198	**-39**	**-31**	**64**	**4.57**	**4.14**	**131**	**0.003**
-45	-19	58	3.81	3.55			-33	-16	64	4.55	4.13		
-27	-10	67	3.61	3.38			-27	-10	67	4.29	3.93		

⁎ The table shows 3 local maxima >8 mm apart. The maximum of each cluster is plotted in bold. Note that the cluster in the left hemisphere did not reach significance with the contrast *POST*_*ON_left*_−*PRE*_*ON*_. All clusters are shown in Fig. 2, top row, in magenta (*POST*_*ON_left*_*−PRE*_*ON*_) and red color (*POST*_*ON_right*_*−PRE*_*ON*_). EC = eigenvector centrality; *x y z* = coordinates in mm; *T* = *T*-score; *Z* = *Z*-score; *k* = cluster size in voxels (1 voxel = 27 mm^3^); *FDR-*corr = corrected for multiple comparisons using the false discovery rate.

**Table 3:** List of clusters showing an increased EC in the left and right motor cortex with the ‘add’ approach comparing DBS with levodopa medication using the contrast *POST*_*ON*_*−PRE*_*ON*_ combining both EC maps with left and right unilateral STN DBS using a flexible factorial model with *POST*_*ON*_*=POST*_*ON_left*_ *+* *POST*_ON_right_*.

*POST*_*ON*_−*PRE*_*ON*_							(*POST*_*ON*_−*PRE*_*ON*_)−(*POST*_*OFF*_−*PRE*_*OFF*_)						
*x*	*y*	*z*	*T*	*Z*	*k*	*p*_*FDR-corr*_	*x*	*y*	*z*	*T*	*Z*	*k*	*p*_*FDR-corr*_
**30**	**−16**	**67**	**5.04**	**4.49**	**219**	**<0.001**	**45**	**−25**	**52**	**3.77**	**3.51**	**68**	**0.034**
18	−22	67	4.42	4.03			30	−16	67	3.76	3.50		
9	−28	49	4.36	3.98			45	−22	58	3.48	3.27		
**−33**	**−16**	**64**	**4.88**	**4.37**	**112**	**0.008**	**−21**	**−7**	**61**	**4.87**	**4.36**	**68**	**0.034**
−27	−10	67	4.31	3.94			−27	−16	67	3.46	3.25		
−39	−31	64	4.22	3.87			−24	−28	64	3.02	2.87		

*The table shows 3 local maxima >8 mm apart. The maximum of each cluster is plotted in bold. All clusters are also shown in Fig. 4, color coded in green and yellow, top row for the contrast *POST*_*ON*_*−PRE*_*ON*_, and bottom row for the interaction contrast (*POST*_*ON*_−*PRE*_*ON*_)−(*POST*_*OFF*_−*PRE*_*OFF*_). EC = eigenvector centrality; *x y z* = coordinates in mm; *T* = *T*-score; *Z* = *Z*-score; *k* = cluster size in voxels (1 voxel = 27 mm^3^); *FDR*-corr = corrected for multiple comparisons using the false discovery rate.

Note that we also checked for EC decreases comparing DBS to levodopa treatments using the contrasts *PRE*_*ON*_*−POST*_*ON_left*_, *PRE*_*ON*_*−POST*_*ON_right*_, and *PRE*_*ON*_*−POST*_*ON*_ using all three approaches of computing EC. We did not observe significant changes, with the exception of a significant EC decrease in the vicinity of the tip of the electrodes (predominantly using the ‘pos’ approach). Additionally, we were not able to show a significant interaction between both factors *PRE/POST* and *ON/OFF*.

### 3.3 Seed-based correlation analysis

**Fig. 5:**
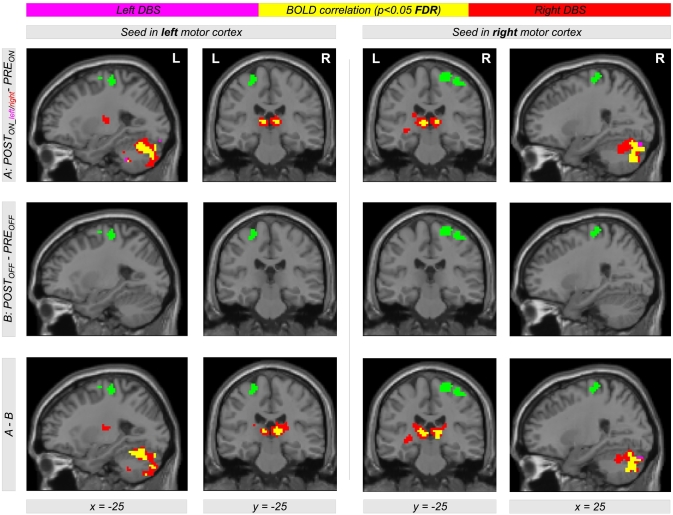
Differential effects of levodopa medication and deep brain stimulation (DBS) in the subthalamic nucleus (STN) on functional connectivity in motor networks affected by Parkinson's disease. The top row shows an increased correlation between BOLD time courses of the motor cortex (green color) with other brain regions within the motor mask during the STN DBS condition (*POST*_*ON_left*_ and *POST*_*ON_right*_) in comparison with the levodopa medication (*PRE*_*ON*_). Using the contrasts *POST*_*ON_left*_*−PRE*_*ON*_ and *POST*_*ON_right*_*−PRE*_*ON*_ (color-coded in magenta and red, respectively; overlap in yellow), the figure shows an increased correlation between the BOLD signal of the motor cortex with the BOLD signal in thalamus and cerebellum in both hemispheres irrespective of using left or right unilateral STN DBS. Interestingly, similar increased correlations were obtained using a seed-region in the left or in the right motor cortex. The second row shows the result for the same analysis comparing both *OFF*-conditions. Note that there are no significant results when using the contrast *POST*_*OFF*_−*PRE*_*OFF*_. The bottom row shows the interaction between both factors *PRE/POST* and *OFF/ON* using the contrast including the difference (*POST*_*ON_left*_*−PRE*_*ON*_)−(*POST*_*OFF*_−*PRE*_*OFF*_) and (*POST*_*ON_right*_−*PRE*_*ON*_)−(*POST*_*OFF*_−*PRE*_*OFF*_) (color-coded in magenta and red, respectively; overlap in yellow). The interaction result (bottom row) shows the same result as the pattern obtained with the treatment-related difference between STN DBS and levodopa (top row). This indicates that our observations are not just due to microlesion or other potential *PRE-POST*-effects. All results are significant using *p* < 0.05 after correction for multiple comparisons using the false discovery rate (FDR) approach.

To investigate which brain regions are stronger connected to the left and right motor cortex leading to increased EC when comparing levodopa medication and DBS treatment, seed-based connectivity analyses were performed. Here, we used seed regions in the left and right motor cortex obtained by EC differences using the contrast *POST*_*ON*_―*PRE*_*ON*_ (see regions color-coded in yellow in Fig. 4, and color-coded in green in Fig. 5). For a comparison between all resulting correlation maps, the statistical analysis was performed with the general linear model using a flexible factorial design described above. Thus, we used the same contrasts for the analysis of the correlation maps as used for investigating EC differences. Comparing DBS with levodopa treatment using the contrasts *POST*_*ON_left*_−*PRE*_*ON*_ and *POST*_*ON_right*_−*PRE*_*ON*_, we received bilateral increased correlation between BOLD time courses of motor and cerebellar regions, and between BOLD time courses of the motor cortex and the thalamus (see Fig. 5, top row, color coded in magenta with left STN DBS, color-coded in red using right STN DBS). Interestingly, this increased correlation was observed in both hemispheres irrespective of using unilateral left or right STN DBS (see overlap color-coded in yellow in Fig. 5). We also observed the same results when using the left or the right motor cortex as seed region (compare the left two columns with the right two columns of Fig. 5, see also Fig. S3, Fig. S4 in the supplement with using the left and the right motor cortex as seed-region, respectively).

To check the influence of microlesion and/or other *PRE-POST*-effects, we also compared the correlation maps in the *OFF*-state. However, we did not find any significant increase using the contrast *POST*_*OFF*_*−PRE*_*OFF*_ (Fig. 5, middle row). The bottom row of Fig. 5 shows the interaction between both factors *treatment method* (*PRE/POST*) and *treatment state* (*OFF/ON*) using the contrast including the difference of difference (*POST*_*ON_left*_−*PRE*_*ON*_)−(*POST*_*OFF*_−*PRE*_*OFF*_) and (*POST*_*ON_right*_*−PRE*_*ON*_)−(*POST*_*OFF*_*−PRE*_*OFF*_) (color-coded in magenta and red, respectively; overlap in yellow). Here, we see the same regions as shown in the top row of Fig. 5, indicating that the observed increase of correlation is due to the treatment change and not induced by microlesion or other *PRE-POST*-effects. All results are significant with *p* < 0.05 using correction for multiple comparisons using the FDR approach.

### 3.4 Motion effects

The analysis of head motion during MR scanning yielded overall very subtle effects. For all subjects and sessions, the mean FD was below 0.7 mm (see Table S1 in the supplement). When disregarding the 5% largest FD values, the maximum remaining FD was <2 mm, which is well below the nominal voxel dimension of our fMRI study (see Table S2). Only 12 out of 12,935 frames from the entire study (i.e. 13 patients × 5 sessions × 199 image volumes) indicated single head movements by >2 mm, corresponding to <0.1% (see Table S1 for details). Moreover, there were no consistent differences in the motion parameters between the different conditions. We note that we exclusively recruited patients of akinetic-rigid type in the current study but no tremor-dominant patients, which explains why motion-related bias was not an issue in this particular cohort.

## 4 Discussion

We investigated brain connectivity alterations in PD patients when comparing two conventional treatments―oral levodopa medication and STN DBS. This is, to the best of our knowledge, the first study directly comparing these two treatments in a single patient cohort. EC mapping and subsequent seed-based correlation analyses demonstrated different changes in connectivity for both treatment modalities, while both led to similar motor improvements according to analyses of motor symptoms (UPDRS-III). Overall, we found that the treatment effect due to STN DBS, as compared to levodopa, was associated with increased connectivity inside the cerebello-thalamic-cortical network. Specifically, both the left and the right motor cortex showed increased interconnectedness in the STN DBS state. These regions in turn showed increased connectivity with the thalamus and the cerebellum in the seed-based analysis. Generally, an increased EC in a brain region means increased connectivity to other regions that have a high degree of interconnectedness themselves. Consequently, regions showing an increased EC obtain a more dominant role in the brain's functional networks (Lohmann et al., 2010). In contrast to other connectivity approaches using specific hypotheses by selecting a few regions (Friston et al., 1997; Friston et al., 2003), EC mapping can be used in a data-driven way for a large number of voxels. The method detects the main network hubs or regions of main connectivity changes. Such regions may then be selected for subsequent connectivity analyses of the network structure.

According to the previous literature, resting state brain connectivity is differently modulated by levodopa and STN DBS. Levodopa, by increasing dopamine availability, induces widespread changes in brain functional connectivity both within and outside the motor network (Tahmasian et al., 2015). Notably, Kelly et al. (2009) confirmed the widespread levodopa effect even in healthy subjects, showing in a double-blind placebo-controlled study that its administration modulates resting state connectivity both in cognitive and motor striatal networks. As regard STN DBS, at first, a simple model was proposed where STN inhibition, induced by high frequency electrical stimulation, leads to reduced glutamatergic output and consequent facilitation of the basal ganglia direct pathway (Hamani et al., 2004). Then, more recent studies proposed that STN DBS efficacy is mediated by a complex modulation of brain networks, for example by means of antidromic activation of input structures (Ashkan et al., 2017; Li et al., 2012). Li et al. (2012) clearly demonstrated in a rat model that STN DBS generates stochastic spikes that travel antidromically along the axons and directly influence the spiking probability of the input cortical regions. We believe that STN DBS acts through a complex combination of these proposed mechanisms as reflected by the connectivity changes detected by our study comparing DBS and levodopa. In the literature, DBS related connectivity changes are rarely reported with resting state fMRI in PD patients. A recent paper assessed the impact of DBS on effective connectivity using dynamic causal modeling (Kahan et al., 2014). Similar to our study, twelve PD patients were scanned with and without DBS using a Siemens 1.5T MRI system. Comparing different models with Bayesian model selection, highest evidence was received with a model that modulates all the major components of the motor cortico-striato-thalamo-cortical loop. Interestingly, the strength of thalamo-cortical pathways was increased that is in line with our findings. As aforementioned, Horn et al. (2017) found that the anticorrelation between the STN and primary motor cortex studied in a large sample of healthy subjects was predictive of a better treatment response to DBS in PD patients. However, since we performed our analysis in PD patients implanted with STN DBS, we had to exclude the brain regions surrounding the electrode from the analysis and consequently the study of STN functional connectivity was not possible.

Comparing between STN DBS treatment and levodopa medication, we observed an increased EC in both the left and the right motor cortex suggesting a treatment effect on the degree of interconnectedness of these regions. Interestingly, this EC increase in the left and right motor cortex was obtained with both unilateral stimulation of the left and the right STN. Subsequent correlation analyses showed connectivity changes between the left and the right motor cortex and thalamus bilaterally―a pathway that is known to be affected in PD patients (Lozano et al., 2002). Remarkably, a very similar pattern of connectivity increase was observed for both hemispheres, that is, left and right STN stimulation yielded similar connectivity results. Note that this finding of bilateral connectivity increase upon unilateral stimulation does not contradict previous work that discussed an ipsilateral increase of brain activity by an increasing blood flow (Ceballos-Baumann, 2003; Lozano et al., 2002). In contrast to this work discussing brain activity, we investigated connectivity changes based on the correlation between BOLD time courses of different brain regions without performing a motor task. As the correlation between time courses is not related to the signal amplitude alone, an increased correlation might be accompanied even with no change of signal amplitude, for instance due to reductions in the phase shift. In this scenario, connectivity increase would be observed without detecting brain activity increase. Several reasons could account for our findings. For example, the bilateral increased cortical interconnectedness in the presence of unilateral stimulation might be mediated by the strong transcallosal transmission between homologue sensorimotor regions (Fling et al., 2013). Additionally, it has been proposed that the antidromic activation of the bilateral cortical afferents to the STN might contribute to the bilateral effect of unilateral stimulation (Knight et al., 2015). Arai et al. (2008) demonstrated, by means of [18F]-fluorodeoxyglucose PET, that the bilateral clinical efficacy of unilateral STN DBS is mediated by ipsilateral thalamic activation and contralateral GPi deactivation that would eventually lead to bilateral cortical activation. In line with these findings, clinical observations of PD patients with unilateral STN DBS reported both contralateral and, to a less extent, ipsilateral and axial improvements in motor performance (Chung et al., 2006; Kumar et al., 1999; Linazasoro et al., 2003; Lizarraga et al., 2016). Further experiments with unilateral DBS would be appreciated to confirm the observed connectivity changes. Of note, the convergence of the three different methodological approaches implemented here to deal with negative correlations in the similarity matrix (i.e. ‘add’, ‘abs’ and ‘pos’) suggests that our findings of EC increase are mainly based on the positive correlations between BOLD time courses. Additionally, we also found a decrease of interconnectedness in the *PRE*_*ON*_−*POST*_*ON*_ comparison, but without evidence for an interaction between both factors *PRE/POST* and *ON/OFF*. Thus, we were not able to disentangle the finding of EC decrease from the susceptibility effects related to insufficient masking of the electrode.

The clinical data show that both levodopa and DBS in the STN led to comparable improvement in motor function, namely clinical symptoms as investigated with the UPDRS-III score. In addition, STN DBS led to a significant reduction of treatment-related motor complications. Hence, we cannot exclude that the qualitative or quantitative changes in the clinical symptoms profile after STN DBS implantation possibly contributed to the observed connectivity difference between STN DBS treatment and levodopa medication. Furthermore, surgery improves clinical symptoms by two mechanisms―firstly the so-called microlesion effect, where a temporary local edema due to electrode implantation leads to transient improvement of clinical symptoms, and secondly the proper stimulation effect (Jech et al., 2012). We have previously shown in *exactly* the same cohort of patients as investigated here that penetration of electrodes was associated with increased EC in the brainstem as shown in Fig. 3c in (Holiga et al., 2015). As our patients were examined soon after surgery and, hence, with still externalized electrodes, the *POST*_*ON*_ condition contains summation of DBS and thee microlesion effect, which affected both post-operative conditions. This explains why the motor improvement with the STN DBS (*POST*_*ON*_*−POST*_*OFF*_) was smaller than the effect of levodopa (*PRE*_*ON*_*−PRE*_*OFF*_) (see Fig. 1). However, this should not be regarded as evidence of lower efficacy of STN DBS in comparison to levodopa but rather a consequence of stimulation setup to avoid overstimulation of the STN. Future studies are needed to evaluate our findings in larger cohorts and, to confirm recent findings, that fMRI may predict treatment success individually in a pre-surgery period (Horn et al., 2017).

## 5 Conclusion

Although both levodopa and DBS led to comparable improvement of behavior/clinical symptoms as measured with the UPDRS-III score, similar therapeutic effects were accompanied with a major difference in brain connectivity between both treatment approaches. EC mapping revealed a major increase of interconnectedness in the motor cortex with using STN DBS compared to levodopa treatment. This increase of EC was accompanied by an increase of connectivity between the motor cortex and the thalamus, and between the motor cortex and cerebellar brain regions. Connectivity alterations due to STN DBS reveal the treatment-specific involvement of different motor circuits as a possible underlying mechanism for therapeutic effect in PD.

The following are the supplementary data related to this article.

**Fig. S2:**
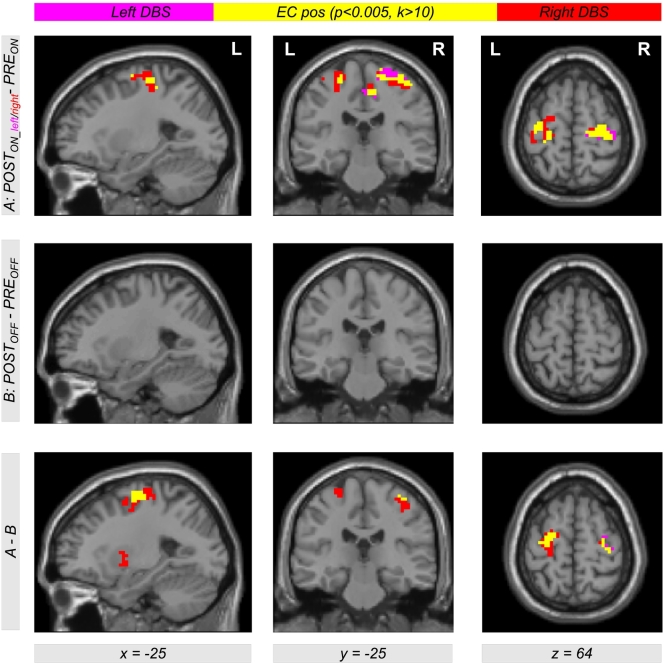
Differential effects of levodopa medication and deep brain stimulation (DBS) in the subthalamic nucleus (STN) on functional connectivity in motor networks affected by Parkinson's disease. The figure has the same content as Fig. 2 using the ‘pos’ approach instead of the ‘add’ approach for computing the EC.

**Fig. S3:**
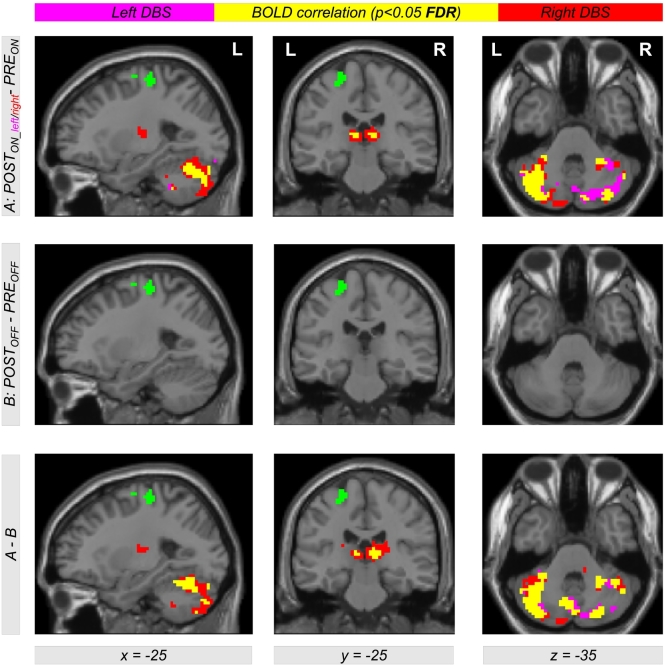
Differences between correlations of BOLD time series between levodopa medication and deep brain stimulation (DBS) in the subthalamic nucleus (STN) in Parkinson's disease. The figure has the same content as Fig. 5 using a seed-region in the left motor cortex including axial slices.

**Fig. S4:**
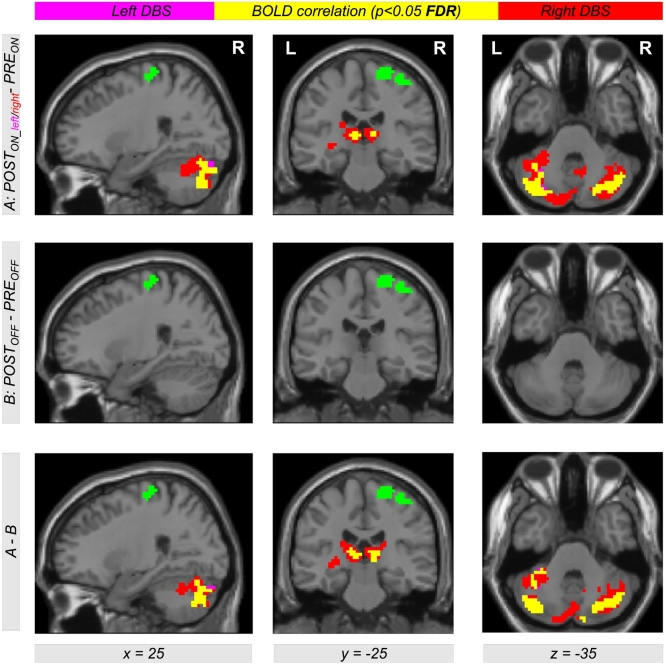
Differences between correlations of BOLD time series between levodopa medication and deep brain stimulation (DBS) in the subthalamic nucleus (STN) in Parkinson's disease. The figure has the same content as Fig. 5 using a seed-region in the right motor cortex including axial slices.

Table S1 Characterization of the subject's motion inside the MR scanner using the mean framewise displacement (FD) and movements with FD > 2 mm obtained by the SPM's translational motion parameters. Table S1

Table S2 Characterization of the subject's motion inside the MR scanner using the maximum framewise displacement (FD) and the maximum FD with disregarding 5% of the largest values (Max95) obtained by the SPM's translational motion parameters. Table S2

Table S3 Individual STN DBS parameters and positions of the active (−) contact with respect to the lateral wall of the third ventricle (x-coordinate) and the mid-commissural point (y-, z-coordinate) within the STN. Bipolar mode was used for stimulation with a reference in contact 3(+) of the 3389 electrode (Medtronic, MN) in all patients. Table S3
