# Supplementary material for: Brain connectivity changes when comparing effects of subthalamic deep brain stimulation with levodopa treatment in Parkinson's disease
Source: Neuroimage Clin. 2018 May 9;19:1025–35. doi: 10.1016/j.nicl.2018.05.006 (PMC6051673; doi:10.1016/j.nicl.2018.05.006)
Supplement: Table S1 — Characterization of the subject's motion inside the MR scanner using the mean framewise displacement (FD) and movements with FD > 2 mm obtained by the SPM's translational motion parameters. [file mmc1.docx]

**Table S1.** Characterization of the subject’s motion inside the MR scanner using the mean framewise displacement (FD) and movements with FD>2 mm obtained by the SPM’s translational motion parameters

|  | Mean FD in mm | | | | | # of movements FD > 2 mm | | | | |
| --- | --- | --- | --- | --- | --- | --- | --- | --- | --- | --- |
| *Subject* | *PRE_OFF_* | *PRE_ON_* | *POST_OFF_* | *POST_ON_left_* | *POST_ON_right_* | *PRE_OFF_* | *PRE_ON_* | *POST_OFF_* | *POST_ON_left_* | *POST_ON_right_* |
| 1 | 0.07 | 0.28 | 0.06 | 0.13 | 0.22 | 0 | 0 | 0 | 0 | 0 |
| 2 | 0.07 | 0.09 | 0.10 | 0.12 | 0.14 | 0 | 0 | 0 | 0 | 0 |
| 3 | 0.07 | 0.70 | 0.08 | 0.13 | 0.18 | 0 | 8 | 0 | 0 | 0 |
| 4 | 0.06 | 0.14 | 0.16 | 0.14 | 0.08 | 0 | 1 | 0 | 0 | 0 |
| 5 | 0.17 | 0.14 | 0.19 | 0.18 | 0.11 | 0 | 0 | 0 | 0 | 0 |
| 6 | 0.09 | 0.29 | 0.11 | 0.13 | 0.10 | 0 | 0 | 0 | 0 | 0 |
| 7 | 0.11 | 0.07 | 0.06 | 0.06 | 0.05 | 0 | 0 | 0 | 0 | 0 |
| 8 | 0.13 | 0.16 | 0.10 | 0.12 | 0.19 | 0 | 0 | 0 | 0 | 0 |
| 9 | 0.24 | 0.44 | 0.08 | 0.13 | 0.13 | 1 | 0 | 0 | 0 | 0 |
| 10 | 0.11 | 0.10 | 0.06 | 0.11 | 0.07 | 0 | 0 | 0 | 0 | 0 |
| 11 | 0.15 | 0.25 | 0.12 | 0.13 | 0.10 | 0 | 0 | 0 | 0 | 0 |
| 12 | 0.03 | 0.05 | 0.04 | 0.14 | 0.04 | 0 | 0 | 0 | 2 | 0 |
| 13 | 0.17 | 0.15 | 0.08 | 0.15 | 0.16 | 0 | 0 | 0 | 0 | 0 |
