# Supplementary material for: Brain connectivity changes when comparing effects of subthalamic deep brain stimulation with levodopa treatment in Parkinson's disease
Source: Neuroimage Clin. 2018 May 9;19:1025–35. doi: 10.1016/j.nicl.2018.05.006 (PMC6051673; doi:10.1016/j.nicl.2018.05.006)
Supplement: Table S2 — Characterization of the subject's motion inside the MR scanner using the maximum framewise displacement (FD) and the maximum FD with disregarding 5% of the largest values (Max95) obtained by the SPM's translational motion parameters. [file mmc2.docx]

**Table S2.** Characterization of the subject’s motion inside the MR scanner using the maximum framewise displacement (FD) and the maximum FD with disregarding five percent of the largest values (Max95) obtained by the SPM’s translational motion parameters

|  | Maximum FD in mm | | | | | Max95 FD in mm | | | | |
| --- | --- | --- | --- | --- | --- | --- | --- | --- | --- | --- |
| *Subject* | *PRE_OFF_* | *PRE_ON_* | *POST_OFF_* | *POST_ON_left_* | *POST_ON_right_* | *PRE_OFF_* | *PRE_ON_* | *POST_OFF_* | *POST_ON_left_* | *POST_ON_right_* |
| 1 | 0.33 | 1.80 | 0.34 | 1.02 | 0.87 | 0.14 | 0.85 | 0.14 | 0.30 | 0.56 |
| 2 | 0.81 | 0.34 | 1.03 | 1.20 | 1.27 | 0.19 | 0.20 | 0.25 | 0.29 | 0.34 |
| 3 | 0.34 | 3.88 | 0.24 | 0.77 | 0.91 | 0.16 | 1.87 | 0.16 | 0.33 | 0.41 |
| 4 | 0.62 | 2.77 | 1.16 | 0.98 | 0.57 | 0.13 | 0.36 | 0.35 | 0.29 | 0.18 |
| 5 | 1.08 | 0.67 | 0.42 | 0.52 | 0.66 | 0.32 | 0.34 | 0.36 | 0.38 | 0.31 |
| 6 | 1.00 | 1.35 | 1.04 | 1.00 | 0.96 | 0.22 | 0.84 | 0.26 | 0.30 | 0.27 |
| 7 | 0.65 | 0.36 | 0.46 | 0.67 | 0.56 | 0.24 | 0.20 | 0.16 | 0.13 | 0.13 |
| 8 | 0.73 | 0.63 | 0.45 | 1.05 | 1.27 | 0.31 | 0.39 | 0.23 | 0.28 | 0.51 |
| 9 | 2.26 | 1.89 | 0.48 | 1.03 | 0.78 | 0.57 | 1.26 | 0.19 | 0.31 | 0.31 |
| 10 | 0.85 | 0.42 | 0.34 | 1.10 | 0.42 | 0.30 | 0.24 | 0.12 | 0.24 | 0.16 |
| 11 | 1.47 | 1.02 | 0.98 | 0.86 | 0.76 | 0.56 | 0.51 | 0.26 | 0.27 | 0.25 |
| 12 | 0.28 | 0.34 | 0.79 | 5.35 | 0.34 | 0.07 | 0.10 | 0.10 | 0.70 | 0.16 |
| 13 | 0.85 | 0.77 | 0.35 | 0.64 | 0.76 | 0.44 | 0.40 | 0.21 | 0.37 | 0.40 |
