# Supplementary material for: Brain connectivity changes when comparing effects of subthalamic deep brain stimulation with levodopa treatment in Parkinson's disease
Source: Neuroimage Clin. 2018 May 9;19:1025–35. doi: 10.1016/j.nicl.2018.05.006 (PMC6051673; doi:10.1016/j.nicl.2018.05.006)
Supplement: Table S3 — Individual STN DBS parameters and positions of the active (−) contact with respect to the lateral wall of the third ventricle (x-coordinate) and the mid-commissural point (y-, z-coordinate) within the STN. Bipolar mode was used for stimulation with a reference in contact 3(+) of the 3389 electrode (Medtronic, MN) in all patients. [file mmc3.docx]

**Table S3.** Individual STN DBS parameters and positions of the active (-) contact with respect to the lateral wall of the third ventricle (x-coordinate) and the mid-commissural point (y-, z-coordinate) within the STN. Bipolar mode was used for stimulation with a reference in contact 3(+) of the 3389 electrode (Medtronic, MN) in all patients.

|  | DBS parameters | | | | active (-) contact right in mm | | | active (-) contact left in mm | | |
| --- | --- | --- | --- | --- | --- | --- | --- | --- | --- | --- |
| ID | amplitude R in V | amplitude L in V | pulse width in μs | Frequency in Hz | x | y | z | x | y | z |
| 1 | 2,5 | 3.0 | 60 | 130 | 7.6 | -3.2 | -2.6 | 5.5 | -3.9 | -4.2 |
| 2 | 2.8 | 2.8 | 60 | 130 | 10.5 | 0.5 | -3.3 | 8.9 | -2.8 | -1.0 |
| 3 | 2.2 | 2.2 | 60 | 130 | 8.1 | -3.3 | -5.8 | 9.2 | -3.3 | -5.0 |
| 4 | 2.3 | 3.0 | 60 | 130 | 11.5 | 0.6 | -4.3 | 7.8 | -5.1 | -4.7 |
| 5 | 2.5 | 3.5 | 60 | 130 | 8.4 | -2.2 | -6.9 | 9.2 | -2.3 | -3.8 |
| 6 | 2.2 | 2.2 | 60 | 130 | 7.2 | -0.8 | -6.1 | 7.3 | -2.6 | -5.3 |
| 7 | 2.5 | 2.5 | 60 | 130 | 9.2 | -0.1 | -4.0 | 10.9 | -2.2 | -2.2 |
| 8 | 1.5 | 2.0 | 60 | 130 | 10.2 | -4.9 | -5.6 | 9.2 | -1.8 | -4.9 |
| 9 | 2.6 | 2.3 | 60 | 130 | 8.4 | -2.8 | -6.7 | 9.6 | -2.8 | -6.1 |
| 10 | 2.9 | 2.9 | 60 | 130 | 9.1 | -1.6 | -4.7 | 8.3 | -1.2 | -3.7 |
| 11 | 3.0 | 3.0 | 60 | 130 | 10.9 | -3.3 | -4.9 | 9.4 | -3.3 | -3.7 |
| 12 | 3.0 | 3.0 | 60 | 130 | 9.3 | -2.6 | -5.0 | 8.3 | -2.3 | -2.8 |
| 13 | 3.0 | 3.0 | 60 | 130 | 9.9 | -2.4 | -6.0 | 9.0 | -1.8 | -4.8 |
